# Imaging different cell populations in the mouse olfactory bulb using the genetically encoded voltage indicator ArcLight

**DOI:** 10.1117/1.NPh.11.3.033402

**Published:** 2024-01-17

**Authors:** Lee Min Leong, Douglas A. Storace

**Affiliations:** a Florida State University, Department of Biological Science, Tallahassee, Florida, United States; b Florida State University, Program in Neuroscience, Tallahassee, Florida, United States; c Florida State University, Institute of Molecular Biophysics, Tallahassee, Florida, United States

**Keywords:** optical imaging, genetically encoded voltage indicator, ArcLight, fluorescent protein, olfactory bulb

## Abstract

Genetically encoded voltage indicators (GEVIs) are protein-based optical sensors that allow for measurements from genetically defined populations of neurons. Although *in vivo* imaging in the mammalian brain with early generation GEVIs was difficult due to poor membrane expression and low signal-to-noise ratio, newer and more sensitive GEVIs have begun to make them useful for answering fundamental questions in neuroscience. We discuss principles of imaging using GEVIs and genetically encoded calcium indicators, both useful tools for *in vivo* imaging of neuronal activity, and review some of the recent mechanistic advances that have led to GEVI improvements. We provide an overview of the mouse olfactory bulb (OB) and discuss recent studies using the GEVI ArcLight to study different cell types within the bulb using both widefield and two-photon microscopy. Specific emphasis is placed on using GEVIs to begin to study the principles of concentration coding in the OB, how to interpret the optical signals from population measurements in the *in vivo* brain, and future developments that will push the field forward.

Techniques that allow for monitoring the activity of genetically heterogeneous populations of neurons would facilitate our ability to understand the brain. First reported more than 70 years ago, optical measurements of brain activity offered a potentially powerful answer to this challenging goal.^1^^–^^4^ The persistent efforts of pioneering neuroscientists and the broader community have yielded remarkable improvements to the hardware and sensors required to perform imaging in the brain. Thanks to these advances, the ability to use points of light to signal the activity of nerve cells, imagined nearly a century ago, is now a commonly used technique in neuroscience.^5^ Here we discuss some of the techniques used to measure neural activity in neurons and review recent experiments using protein-based sensors of voltage to study the activity of different cell populations in the mouse olfactory bulb (OB).

## 1 Organic Dyes Versus Genetically Encoded Sensors

Imaging experiments using voltage and calcium dyes have undergone rapid and extensive development and improvements since they were first described in 1968 (8-anilinonaphthalene-1-sulfonic acid) and 1975 (arsenazo III), respectively.^1^^,^^6^^,^^7^ Since then, the dye toolkit has undergone extensive development resulting in improvements leading to reduced phototoxicity, improved kinetics, and signal-to-noise ratio.^7^^–^^12^ For example, the voltage sensitive dyes di-4-ANEPPS and ANNINE-6plus have time constants on the order of $10^{-3}$ to $10^{-9}\;s$, and linear relationships with voltage sensitivity that yield fractional changes >8% ΔF/F/100  mV.^13^^–^^18^

However, two major challenges of using organic dyes are that they must be physically introduced into the preparation, and they label all cells approximately equally.^19^^,^^20^ The lack of cell-specificity challenged efforts to understand the functional contributions of different cell types, except in exceptional cases where a dye can be anatomically restricted.^21^^–^^29^

A class of protein-based optical sensors have been developed as an alternative to organic dyes that allow for neuron-type-specific labeling, which can be used to determine the role of different neuron types within and across brain areas.^30^^–^^32^ This review primarily focuses on sensor variants in which the dye has been replaced by an optical reporter (e.g., a fluorescent protein) fused to a sensing element (e.g., a voltage or calcium sensing domain).^20^^,^^33^^,^^34^ However, other approaches incorporate a hybrid approach where a genetically encoded protein interfaces with an externally applied organic dye.^35^^,^^36^

The gene encoding the combined protein can be introduced into specific cell types using different promoters, viral vectors, and transgenic mice. Many different kinds of genetically encoded indicators have recently become available that report different cellular signals and binding of different neurotransmitters.^37^^–^^48^ Our review discusses sensors of voltage and calcium, with a specific focus on the use of genetically encoded voltage indicators (GEVIs) to study the mammalian OB.

## 2 Genetically Encoded Calcium and Voltage Indicators

Genetically encoded calcium indicators (GECIs) are protein-based sensors that report changes in intracellular calcium.^33^ Calcium imaging is often used as a proxy for changes in spiking activity because action potentials cause an increase in intracellular calcium. However, intracellular calcium dynamics are shaped by other sources, including subthreshold depolarization,^49^^,^^50^ through ligand-gated receptors,^51^^,^^52^ intracellular sequestration,^53^^–^^55^ and dendritic coincidence detection.^16^ Intracellular calcium dynamics are also substantially slower than voltage changes and cannot perfectly recapitulate action potential activity except at lower rates of spiking activity.^56^^–^^58^ The biophysical properties of genetically encoded indicators can further complicate the interpretation of an optical measurement. The speed in which a GECI transforms changes in intracellular calcium into fluorescence changes can contribute additional temporal filtering,^59^^–^^62^ and in some cases, the kinetics have calcium concentration-dependent nonlinearities.^63^^,^^64^ Many GECIs also exhibit nonlinearities in their relationship between calcium binding and fluorescence changes with Hill coefficients (which describes the degree of cooperativity in ligand–receptor binding) as great as 3.^59^^,^^60^^,^^62^^,^^64^^,^^65^ Each GECI has a different calcium affinity, which defines the center of the calcium range they can detect.^66^ For sensors with linear relationships, this affinity has a straightforward impact on the interpretation of the calcium signals; the same absolute change in calcium will evoke the same magnitude fluorescence change. The interpretation of a nonlinear GECI measurement is more complex because the same change in intracellular calcium could result in different fluorescence changes depending on the calcium concentration. In principle, measurements could be restricted to the linear concentration range of a GECI, although this is difficult or impractical to measure in most preparations. However, ground truth experiments that combined intracellular electrode recordings and calcium imaging have shown that some GECIs have approximately linear relationships between action potential activity and calcium signals across some spike rates.^65^^,^^67^ Since their introduction 25 years ago, engineering efforts have improved GECI sensitivity, brightness, and speed.^32^^,^^65^^,^^66^^,^^68^^,^^69^ Importantly, the growing toolkit of widely available viral vectors and transgenic reporter mice have simplified their use.^70^^,^^71^ These developments have revolutionized neuroscience by facilitating the ability to measure changes in neural signaling in a variety of preparations.

If the experimental goal is to measure subthreshold events or changes in spiking activity, direct optical measurements of voltage changes would be ideal. GEVIs are protein-based sensors that optically report changes in membrane potential.^20^^,^^34^ GEVI development has been slower in contrast with GECIs, which reflects the unique challenges involved in measuring cellular voltage changes. GEVIs must be sufficiently fast to detect voltage changes that can occur across millisecond timescales, and their expression must be restricted to the membrane because nonmembrane expression will reduce the signal-to-noise ratio by contributing fluorescence that is not voltage-dependent.^19^^,^^72^ Several biophysical properties of a GEVI are important in evaluating its ability to optically report voltage signals in neural tissue and in the interpretation of its optical signals.^73^^–^^75^ The voltage-sensitivity range of a GEVI determines the absolute voltage changes that will be translated into optical changes.^75^^–^^77^ The sensitivity of a GEVI is defined as the dynamic range of the fluorescence change across its voltage-sensitivity range, which is a key determinant of the signal-to-noise ratio. GEVI brightness is important because relatively few protein molecules can be introduced into the membrane and rapid voltage changes require fast frame rates that limit photon integration time. A sensitive GEVI with very low baseline fluorescence will be difficult to measure due to signal-to-noise issues and may require intense or biologically incompatible illumination levels.^78^ The speed at which voltage changes are transformed into fluorescence changes defines the kinetics of a GEVI. Onset and offset kinetics are typically measured in response to a voltage step and reported as the time to reach its peak fluorescence change or decay back to its baseline. A highly sensitive GEVI with a slow onset will be unable to reach its peak fluorescence change in response to a fast voltage change.^79^ The fluorescence signal from a GEVI with a linear voltage-sensitivity slope centered around −20  mV with submillisecond kinetics will include similar proportions of subthreshold and action potential spiking activity. In comparison, the fluorescence from a GEVI with similar voltage sensitivity but slower onset dynamics will likely reflect more subthreshold voltage signals.^80^ Shifting the voltage sensitivity of a GEVI to a more positive range will yield fluorescence signals that primarily report action potential activity.^73^^,^^77^ In principle, these properties could be optimized to engineer highly specific and targeted probes.^74^^,^^76^^,^^81^^,^^82^

## 3 Principles of GEVI Mechanisms of Action

The first generation of GEVIs based on fusions between green fluorescent protein (GFP) and the voltage-sensing domain (VSD) of potassium or sodium channels did not function well in mammalian cells due to their poor membrane expression.^31^^,^^72^^,^^83^^–^^85^ The discovery and integration of the voltage-sensing phosphatase of *Ciona intestinalis* (sea squirt) with various fluorescent proteins was a milestone in GEVI development.^86^^,^^87^ In this sensor design, optical changes are believed to occur via voltage-dependent movement of the S4 transmembrane domain, which causes movement of the fluorescent protein via its linking element with the VSD.^88^ The first design strategy using the *Ciona* *intestinalis* VSD utilized Förster resonance energy transfer (FRET) fluorescent protein pairs that are attached to the N and C terminus of the VSD, respectively; changes in membrane potential are reported as FRET changes.^86^^,^^89^^,^^90^ Another significant milestone in GEVI development was the serendipitous discovery that modifications to the fluorescent protein and its linking position with the VSD can modulate GEVI sensitivity. In a GEVI utilizing the *Ciona intestinalis* VSD with the eGFP variant super ecliptic pHluorin, a point mutation (A227D) resulted in a large increase in sensitivity.^91^ Further tuning of this mutation along the linking position between the VSD and fluorescent protein yielded the GEVI ArcLight, which exhibited a ∼25-fold increase in sensitivity in comparison with its unmutated version.^91^ ArcLight has a sigmoidal voltage sensitivity relationship with a $v_{1/2}$ around −26  mV. In response to a 100 mV depolarizing step, ArcLight responds with a sensitivity of ∼40% ΔF/F, which can be best fit with a double exponential curve with fast onset and offset components of ∼10 and 50 ms, respectively.^34^^,^^63^^,^^91^ In practice, these dynamics are sufficiently fast to solve individual action potentials at upward of 60 Hz.^73^^,^^91^^,^^92^ Although ArcLight is based on a variant of eGFP, its brightness will be a complex function of many experimental factors including the preparation type and expression level.^91^^,^^93^

Newer GEVIs have incorporated the voltage-gated phosphatase gene discovered in different species with various combinatorial mutations with alternate fluorescent proteins including a circularly permutated GFP inserted into the S3 and S4 loops of the transmembrane segments.^14^^,^^92^^,^^94^^–^^99^ Another developmental strategy has been to incorporate microbial rhodopsins, which make use of the voltage sensitivity and fluorescence of the retinal residing in the opsin protein at the membrane.^78^^,^^100^^,^^101^ A change in membrane potential results in a chemical change that alters the fluorescence of the opsin retinal complex.^102^ The quantum yield of the opsin retinal complex is low, resulting in weak fluorescence that requires very high illumination intensities.^101^^,^^103^ Some of these limitations have been overcome by fusing opsins with fluorescent proteins to form an FRET pair, resulting in substantially brighter fluorescence than the opsin alone.^104^^–^^107^ One hybrid strategy incorporates rhodamine-based voltage dyes with a genetically encoded HaloTag to target the dye to specific neuronal populations. Another strategy based on enzymatic cleavage via photoinduced electron transfer of voltage dyes, where VoltageFluor remains dim until activated porcine liver esterase.^108^^,^^109^ These advances in GEVI engineering have facilitated their use in biological discoveries in different brain regions and preparation types.^106^^,^^110^^–^^116^

## 4 Organization and Circuitry of the Olfactory Bulb

The sense of smell is critical for many animals as it plays a key role in locating danger, food, and mates. In mice, olfactory receptor neurons (ORNs) express one out of a large number of olfactory receptor proteins (∼1000), each with a distinct affinity for an odor.^117^^–^^120^ Different odor-concentration pairings evoke varying degrees of activity across the olfactory receptor population, resulting in a combinatorial code, which is transmitted into the OB.^121^^,^^122^ Each ORN type typically maps to one or two regions of OB neuropil called glomeruli.^123^^–^^126^ Each glomerulus is innervated by the apical dendrites of a specific population of mitral and tufted cells (MTCs), which receive ORN input and project broadly to the rest of the brain.^127^^,^^128^ This input–output transformation is shaped by a complex synaptic network of anatomically and genetically heterogeneous interneurons.^129^^,^^130^ ORNs drive activity to MTCs through feed-forward and lateral pathways via interneurons that surround glomeruli that innervate their parent or neighboring glomeruli.^129^^,^^131^^–^^135^ An additional lateral modulatory circuit is mediated via granule cells that make inhibitory connections on the lateral dendrites of MTCs.^136^^,^^137^ These networks are further shaped by presynaptic modulation of the ORN axon terminals^134^^,^^138^^,^^139^ and cortical feedback from other brain regions.^140^^–^^145^

Defining the functional role(s) of the different populations of neurons within the OB remains an important step in understanding the function of the bulb in olfactory sensory processing.^129^^,^^146^ A recent strategy in addressing this question is to measure and compare the glomerular signals originating from different OB cell types.^67^^,^^147^^–^^149^ Glomerular imaging is powerful because upward of 100 glomeruli can be simultaneously measured from the dorsal surface of the OB, each of which contains the processes of different cell types involved in the input–output transformation for a single olfactory receptor type.^129^^,^^150^^,^^151^ Glomerular measurements to date have reflected the average of different populations of neurons innervating a glomerulus, trading single-cell resolution for a population average. Neural activity measurements of population and single cells can be used to answer different kinds of questions; both have provided critical insight into the logic of olfactory sensory processing.^21^^,^^148^^,^^149^^,^^152^^–^^156^

Glomerular signals have been measured from nonspecific neuronal populations by measuring changes in light reflectance, autofluorescence, and fluorescence from bath applied dyes.^151^^,^^157^^–^^165^ Early efforts to perform cell-specific glomerular imaging were carried out by infusing calcium sensitive organic dyes into the nasal cavity of rodents, which results in the dye being transported to the ORN axon terminals in the OB where they can be imaged.^21^^,^^23^^,^^26^^,^^166^ ORNs have also been imaged using genetically encoded indicators of synaptic vesicle release and calcium.^148^^,^^149^^,^^167^^–^^169^ Glomerular measurements have been carried out from MTCs and two interneuron populations using transgenic mice expressing a GECI under control of a cell-specific promoter, or that express Cre recombinase combined with Cre-dependent adeno associated virus (AAV) transduction or reporter transgenic mice.^67^^,^^148^^,^^149^^,^^152^^,^^170^^–^^174^ Cell-specific glomerular measurements have been important in defining basic coding principles of how odor information is encoded across the OB input–output transformation,^118^^,^^148^^,^^149^^,^^152^^,^^172^^,^^174^^,^^175^ the plasticity of different cell types to perceptual experiences,^168^^,^^176^^–^^178^ and testing models of olfactory sensory processing.^152^^,^^170^

Surprisingly, few of the studies examining sensory coding in the mouse OB have been carried out using voltage imaging.^63^^,^^148^^,^^150^^,^^165^^,^^179^ In addition to providing a direct readout of changes in membrane potential from genetically defined cell populations, voltage imaging can measure fast changes in neural activity that would be otherwise obscured when measured using slower imaging methods, and in principle, should be able to measure depolarizing and hyperpolarizing signals. Together, these advances may eventually provide clearer insight into fast temporal coding, and the nature of the inhibitory circuits within the OB. Here we review recent studies that used the GEVI ArcLight to perform glomerular imaging in the mouse OB.

## 5. ArcLight Measurements from Nonspecific Cell Populations in the Olfactory Bulb

**Fig. 1:**
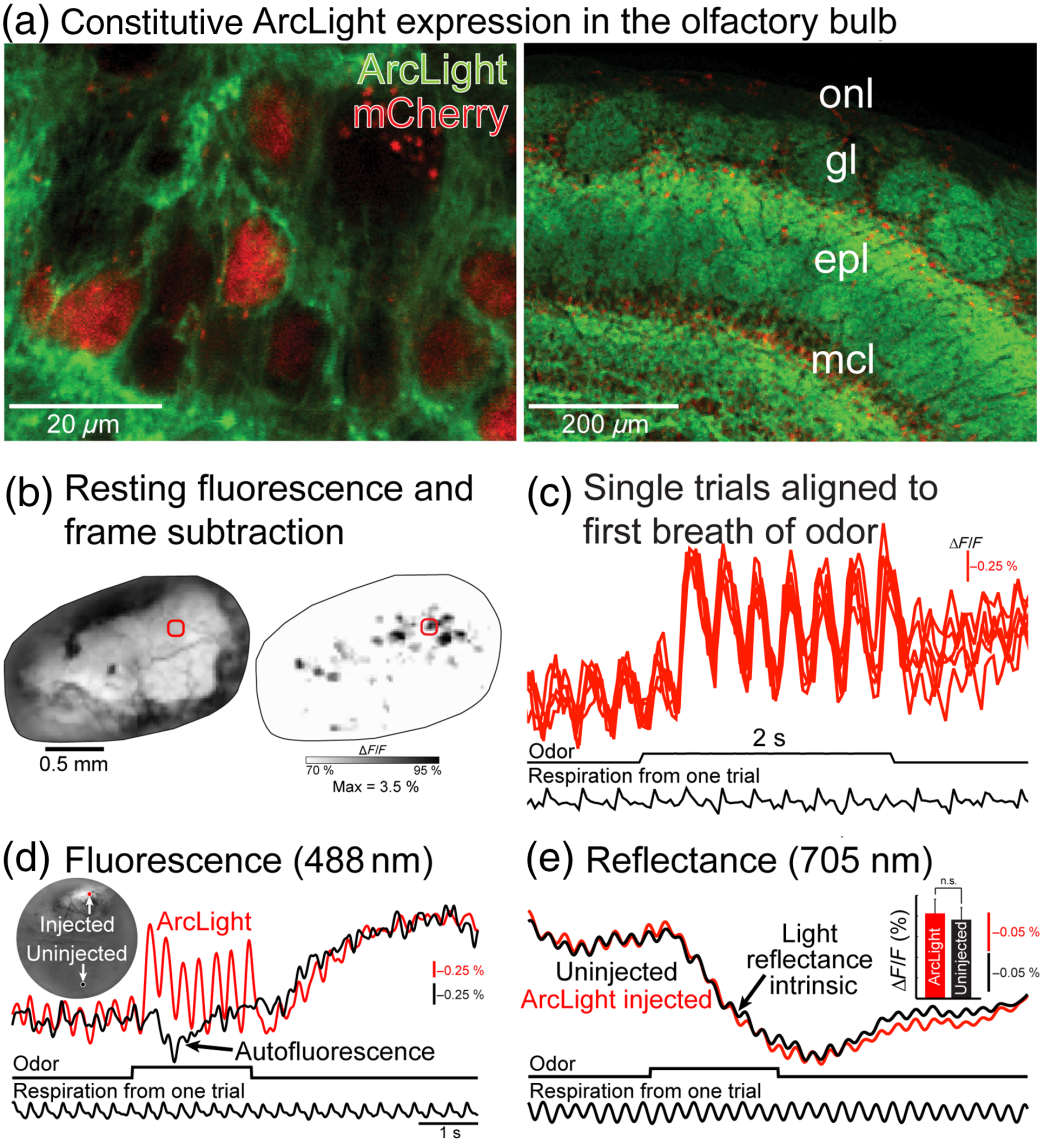
(a) ArcLight expression in the OB from histological images at high (left) and low (right) magnification. (b) ArcLight fluorescence *in vivo* from the OB (left) and a frame subtraction analysis illustrating peaks of activity following odor presentation (right). (c) Single-trial measurements aligned to the onset of respiration from the region of interest in panel (b). (d) Fluorescence measurements from ArcLight injected and uninjected hemibulbs. The inset illustrates the baseline fluorescence and regions of interest. (e) Intrinsic light reflectance measurements from injected and uninjected hemibulbs. The inset illustrates that the intrinsic glomerular signals were not significantly different in the ArcLight injected and uninjected hemibulbs. onl, olfactory nerve layer; gl, glomerular layer; epl, external plexiform layer; and mcl, mitral cell layer.

The first GEVI recordings from the OB were conducted using ArcLight constitutively expressed using an adeno associated virus serotype 1 (AAV1).^63^ The AAV1 was designed to co-express a nuclear localized mCherry fluorescent protein to facilitate visualization of the labeled neurons. Injecting the AAV1 into the mouse OB resulted in membrane localized expression of ArcLight and intracellular expression of mCherry [Fig. 1(a), left]. ArcLight was expressed in a population of OB neurons that included MTCs, consistent with prior reports of the tropism of AAV1 [Fig. 1(a), right].^180^ *In vivo* measurements were made from the dorsal surface of the OB in anesthetized mice using widefield fluorescence microscopy. Odors were presented using a flow-dilution olfactometer and respiration was monitored using a piezosensor placed against the chest of the animal.^181^ Individual glomeruli were not evident in the *in vivo* baseline fluorescence likely because of the presence of diffuse fluorescence originating from ArcLight expressed in neuropil in deeper layers of the OB [Fig. 1(b), left]. Using a frame subtraction analysis in which the imaging frames prior to the odor were subtracted from those acquired during the odor revealed odor-evoked fluorescence changes in glomerular sized regions of interest across the dorsal bulb [Fig. 1(b), right panel]. The fluorescence signals from the glomerular sized regions of interest had a high signal-to-noise ratio that was similar from trial to trial and were often coupled to respiration [Fig. 1(c)]. The resting fluorescence was similarly stable across different trials within a recording session, showing an average decline of ∼1% across individual imaging trials that lasted for ∼8  s (−0.6%±0.02, measurements of baseline fluorescence in 3 to 8 consecutive trials in six different preparations; illumination provided by a 150-W Opti Quip Xenon arc lamp). The fluorescence from the ArcLight expression was substantially brighter than the endogenous autofluorescence present in the uninjected hemibulb, allowing it to overshadow the intrinsic autofluorescence signal that can be measured using the same filter set [Fig. 1(d)].^163^^,^^164^ Furthermore, intrinsic optical signals measured as changes in light reflectance at 705 nm were not statistically different in the ArcLight injected and uninjected hemibulb [Fig. 1(e)]. Therefore, AAV1 transduction of ArcLight yielded sufficiently bright fluorescence for *in vivo* imaging that exhibited minimal photobleaching and phototoxicity.

**Fig. 2:**
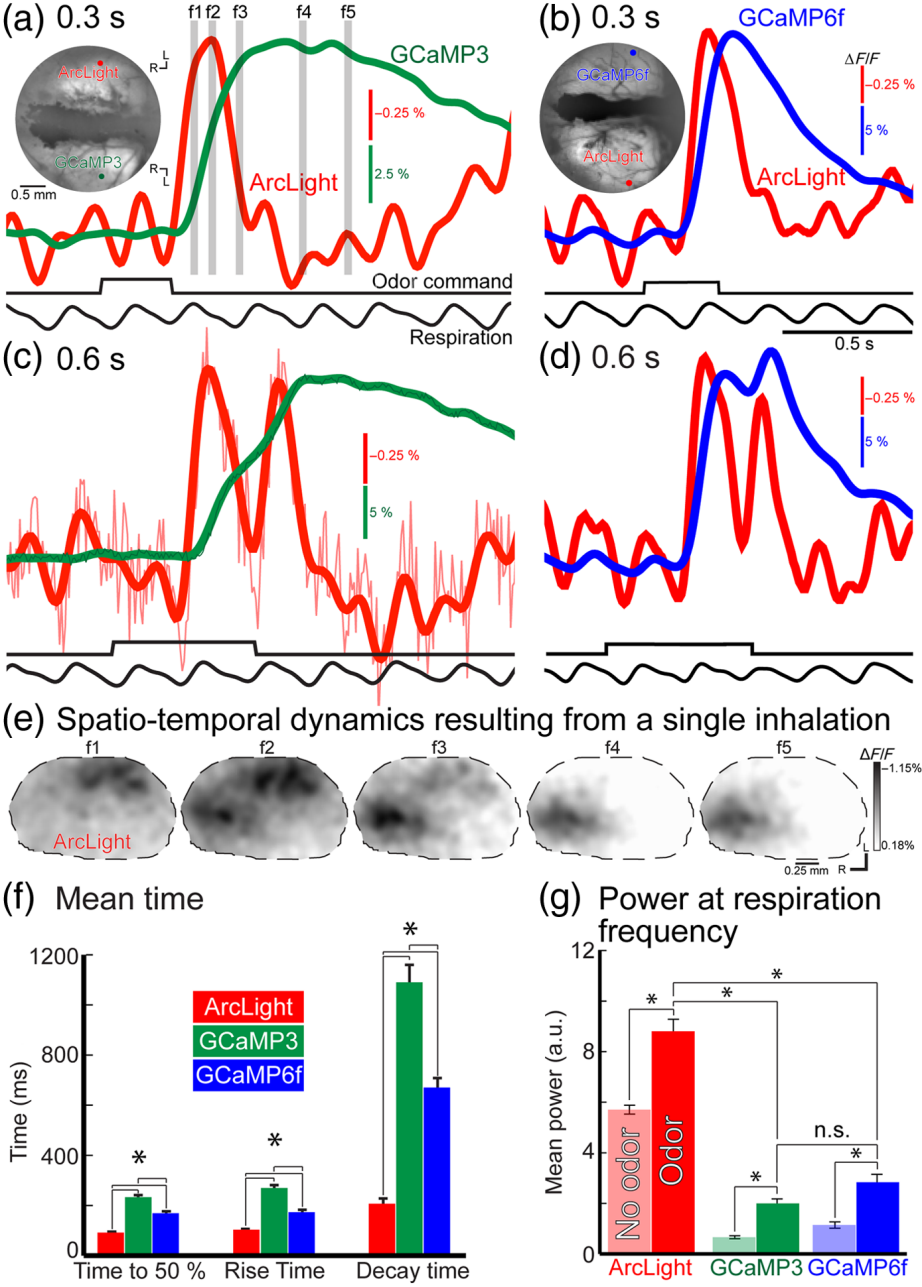
Response to one (a, b) and two (c, d) inhalations of an odor from opposite hemibulbs in two preparations injected with (a, c) ArcLight and GCaMP3 and (b, d) ArcLight and GCaMP6f. The mean fluorescence and regions of interest for the traces for the two preparations are illustrated in the insets. (e) Frame subtraction from the ArcLight injected hemibulb at the timepoints illustrated by the gray bars in panel (a). (f) Temporal properties of the three sensors to one odor inhalation. (g) Power at the respiration frequency for the three sensors during multiple odor inhalations.

In a subset of the ArcLight injected preparations, an AAV1 expressing the GECI GCaMP3 or GCaMP6f was also injected into the opposite hemibulb of the same animal [Figs. 2(a) and 2(b), insets]. Single inspirations of odor resulted in ArcLight and GECI fluorescence changes that could be detected in single trials [Figs. 2(a) and 2(b)]. In trials where the mouse took multiple sniffs of the odor, the ArcLight signal returned most of the way to the baseline between each inspiration; respiratory coupled modulations were less prominent when measured with GECIs [Figs. 2(c) and 2(d), compare red, green, and blue traces]. ArcLight had sufficient temporal resolution to resolve temporal heterogeneity across the glomerular population.^182^ Caudal-lateral glomeruli tended to respond earlier following a single sniff, whereas rostral-medial glomeruli responded more slowly [Fig. 2(e)]. ArcLight signals were smaller than either GECI but had significantly faster onset kinetics, rise times, and decay times [Fig. 2(f)]. To compare the ability of ArcLight and both GECIs to track respiratory coupling, the respiratory frequency was measured using a piezosensor pressed against the mouse’s chest. The signals from ArcLight and both GECIs were normalized to control for differences in signal-to-noise ratio, and their power at the respiratory frequency was measured before and after odor presentation. Odor presentation resulted in a significant increase in power at the respiratory frequency for all three sensors, although ArcLight had a significantly larger change than either GECI [Fig. 2(g)]. The time course of the ArcLight signal is similar to the timing of action potential activity of mitral cells in anesthetized rodents, which spike during inspiration.^183^^–^^185^ The higher temporal resolution of ArcLight may more faithfully represent the time course of respiratory coupled activity in the OB.

## 6 ArcLight in Specific OB Cell Types

**Fig. 3:**
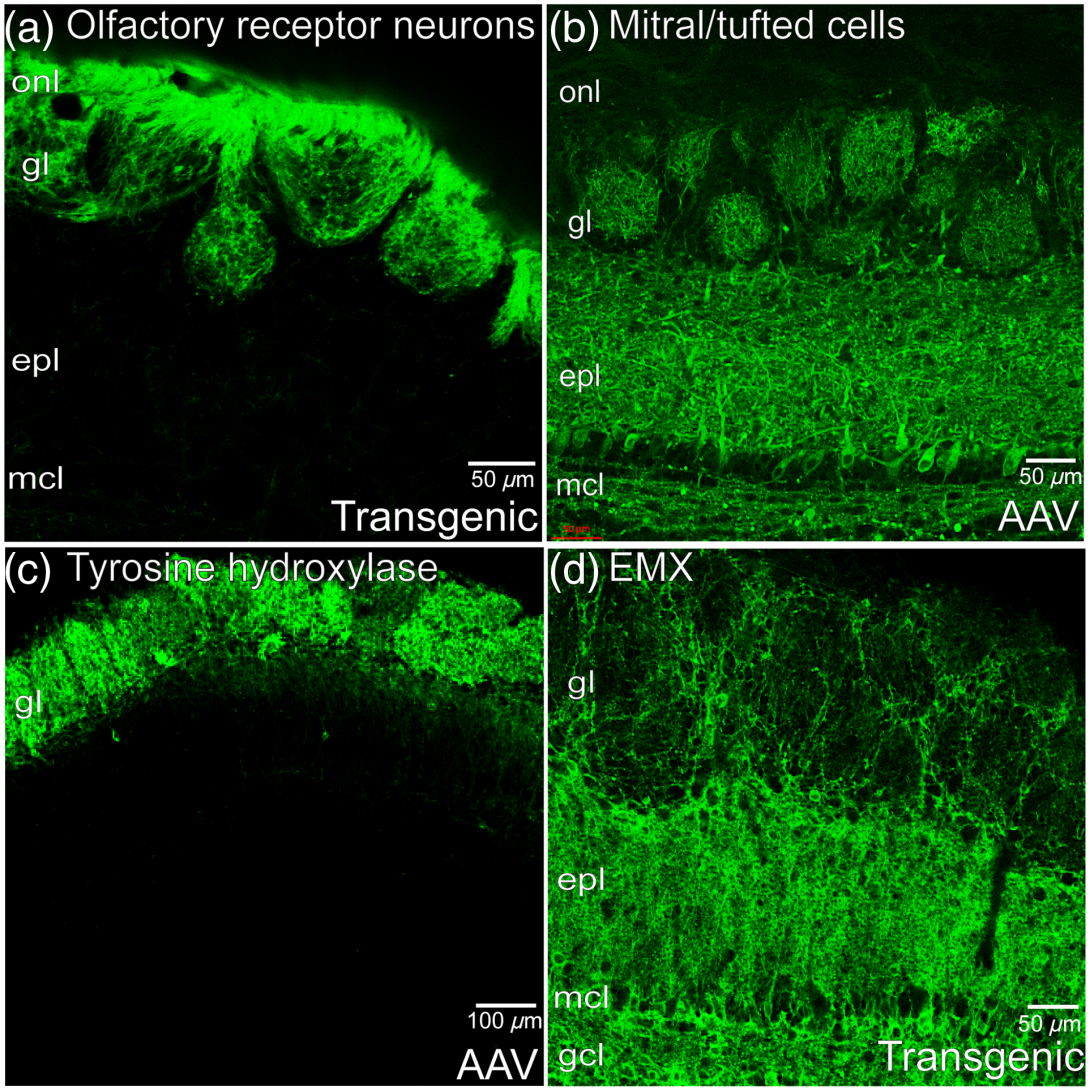
ArcLight expression in (a) ORNs, (b) MTCs, (c) TH interneurons, and (d) Camk2a-EMX interneurons. onl, olfactory nerve layer; gl, glomerular layer; epl, external plexiform layer; mcl, mitral cell layer; and gcl, granule cell layer.

Glomerular measurements from cell-specific populations have been carried out with ArcLight expressed using Cre-dependent AAV transduction and using the Ai86 ArcLight reporter transgenic line, which requires an intersectional breeding strategy to drive co-expression of ArcLight, tTA, and Cre recombinase.^70^^,^^71^^,^^148^^,^^179^ ORN targeting was achieved by first mating Ai86 to the Camk2a-tTA transgenic line.^70^^,^^186^ The resulting offspring that expressed both ArcLight and tTA were subsequently mated to the OMP-Cre transgenic line.^187^ The offspring that expressed all three genes exhibited ArcLight fluorescence in the olfactory nerve layer and glomeruli [Fig. 3(a)].^179^ Selective expression of ArcLight in MTCs was achieved by injecting a Cre-dependent AAV into the OB of protocadherin21-Cre transgenic mice, which resulted in ArcLight fluorescence present in MTC bodies, and their lateral and apical dendrites [Fig. 3(b)].^148^^,^^188^ A Cre-dependent AAV was injected into the OB of TH-Cre transgenic mice to express ArcLight in a population of interneurons. ArcLight fluorescence was present in neurons that surrounded glomeruli along with their associated glomerular processes [Fig. 3(c)].^135^^,^^150^^,^^170^ ArcLight was also expressed in a population of OB interneurons driven by the intersection of the Camk2a and EMX promoters by mating the offspring from pairings between Ai86 and Camk2a-tTA to the EMX-Cre transgenic line. The offspring that expressed all three genes exhibited ArcLight fluorescence in a subset of periglomerular and granule cells^189^ [Fig. 3(d)].

## 7 ArcLight Measurements From Olfactory Receptor Neuron Glomeruli

**Fig. 4:**
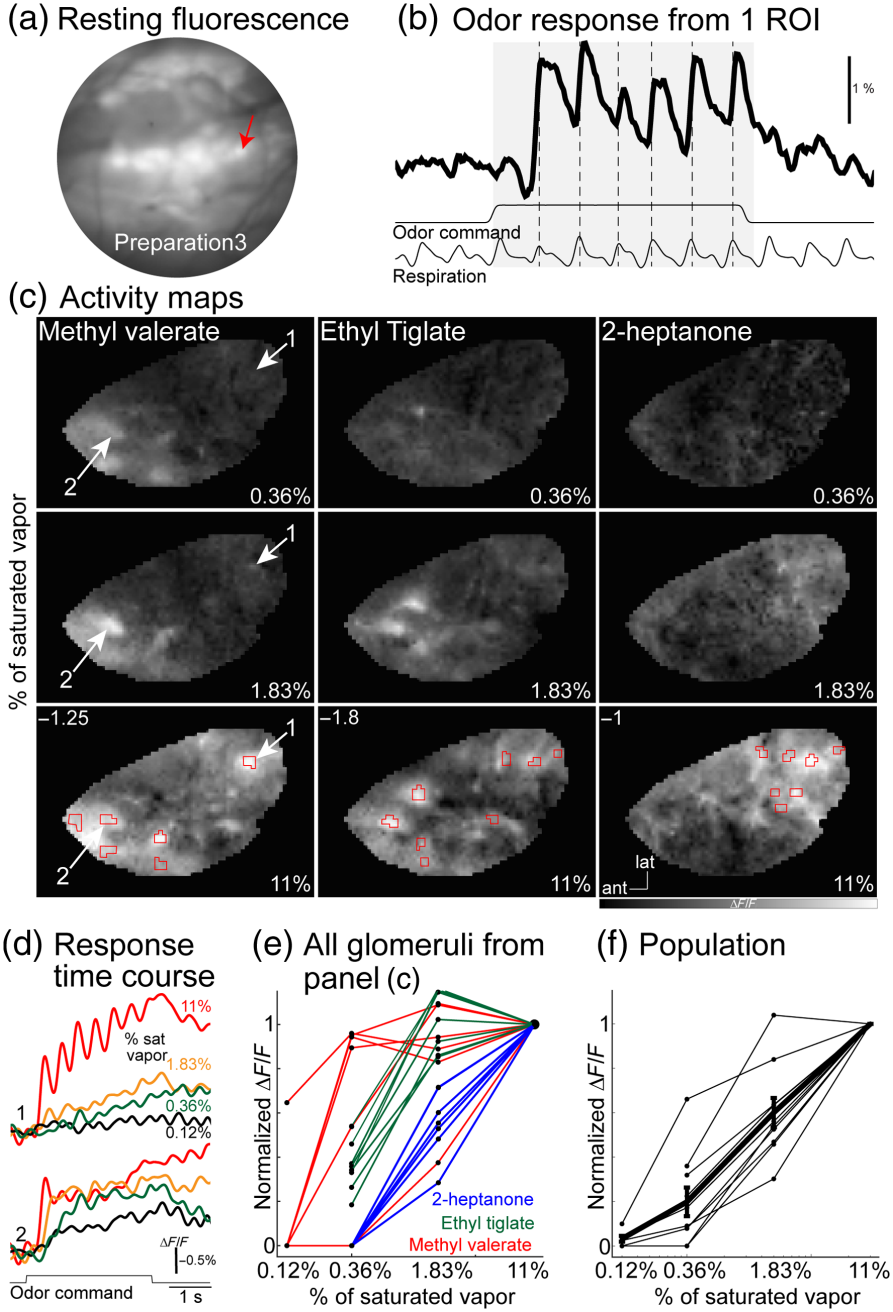
Odor-evoked measurements in OMP-ArcLight transgenic mice. (a) Mean fluorescence *in vivo*. (b) Single-trial odor response from one glomerulus. (c) Frame subtraction maps in response to different odor-concentration pairings. The numbers at the top left of the bottom panels indicate the maximum ΔF/F. (d) Methyl valerate responses at different concentrations in two different glomeruli. (e, f) Normalized response amplitude versus odor concentration for the glomeruli in panel (c) and for a population of preparations.

Olfactory perception must be determined by the pattern of olfactory receptors that are activated by an odor stimulus. Because each ORN glomerulus reflects the input from one receptor type, glomerular measurements provide a readout of how odors are encoded across the receptor population. Widefield fluorescence imaging was used to measure odor-evoked activity from ORNs in anesthetized mice. Individual glomeruli were more clearly visible in the mean fluorescence than in the constitutive AAV experiment, consistent with the expression of ArcLight being restricted to glomeruli [Fig. 4(a)]. Odors evoked fluorescence changes with a high signal-to-noise ratio that were often coupled to each inspiration of the stimulus [Fig. 4(b)]. A frame subtraction analysis revealed that different odors evoked distinct patterns of activity across the glomerular population [Fig. 4(c), columns]. The same odors presented at different concentrations also changed the glomerular patterns; increasing the concentration increased the amplitude of active glomeruli and recruited glomeruli that were not responsive at lower concentrations [Fig. 4(c), rows]. The combination of activated ORNs changes in response to different odors as well as different concentrations of the same odor; representations of odor identity and concentration are confounded at the input to the mouse OB.^21^ The effect of concentration on different ORNs is evident in the fluorescence versus time course signal measured from different glomeruli [Fig. 4(d)]. The steep concentration dependence is quantified by plotting the peak normalized amplitude versus concentration for each glomerulus [Fig. 4(e), rois from panel (c)] and a population of preparations [Fig. 4(f)]. The Hill coefficients of ORN measurements across many glomerulus-odor pairings in a population of preparations ranged between 1 and 2.2, which are consistent with previous ORN concentration response relationships in a variety of different preparations.^21^^,^^118^^,^^190^^–^^195^ These results are consistent with a model in which ORNs are broadly tuned with a range of affinities for different odors.^117^ The steep concentration-response relationships of individual ORNs combined with their narrow dynamic range is the basis of changes in the ORN glomerular maps in response to an odor or concentration change. A threshold concentration of an odor will activate a subset of high affinity ORNs; increasing the concentration saturates high-affinity receptors and recruits inactive low affinity receptors. Importantly, the glomerular ORN voltage imaging results are consistent with similar measurements using other kinds of optical sensors.^21^^,^^23^^,^^118^^,^^148^^,^^167^

## 8 ArcLight Measurements From MTC Glomeruli

Humans and other animals exhibit concentration invariant olfactory perception, which refers to the ability to recognize an odor as the same across concentration changes.^196^^–^^200^ The observation that the patterns of activated ORN glomeruli change in response to different odors, and concentration changes of the same odor has raised the question of where and how concentration invariance is generated in the brain.^117^ Experimental and modeling work supported the possibility that circuitry within the OB could be important for generating more stable olfactory representations.^117^^,^^201^^,^^202^ This possibility was tested using a two-color imaging strategy, in which the signals from the ORNs innervating OB glomeruli were compared with the signals from the MTCs innervating the same glomeruli. In these experiments, the calcium sensitive dye fura dextran was infused into the nasal cavity of Pcdh21-Cre transgenic mice that received a Cre-dependent ArcLight-expressing AAV injection. The resulting mice had their ORN glomeruli selectively labeled with fura dextran and MTCs with ArcLight.^26^^,^^188^ Because fura dextran has an absorption spectra that is distinct from ArcLight, the signals originating from the ORNs and the MTCs innervating the same glomeruli could be differentiated by changing the excitation wavelength.^55^^,^^63^^,^^91^^,^^148^

**Fig. 5:**
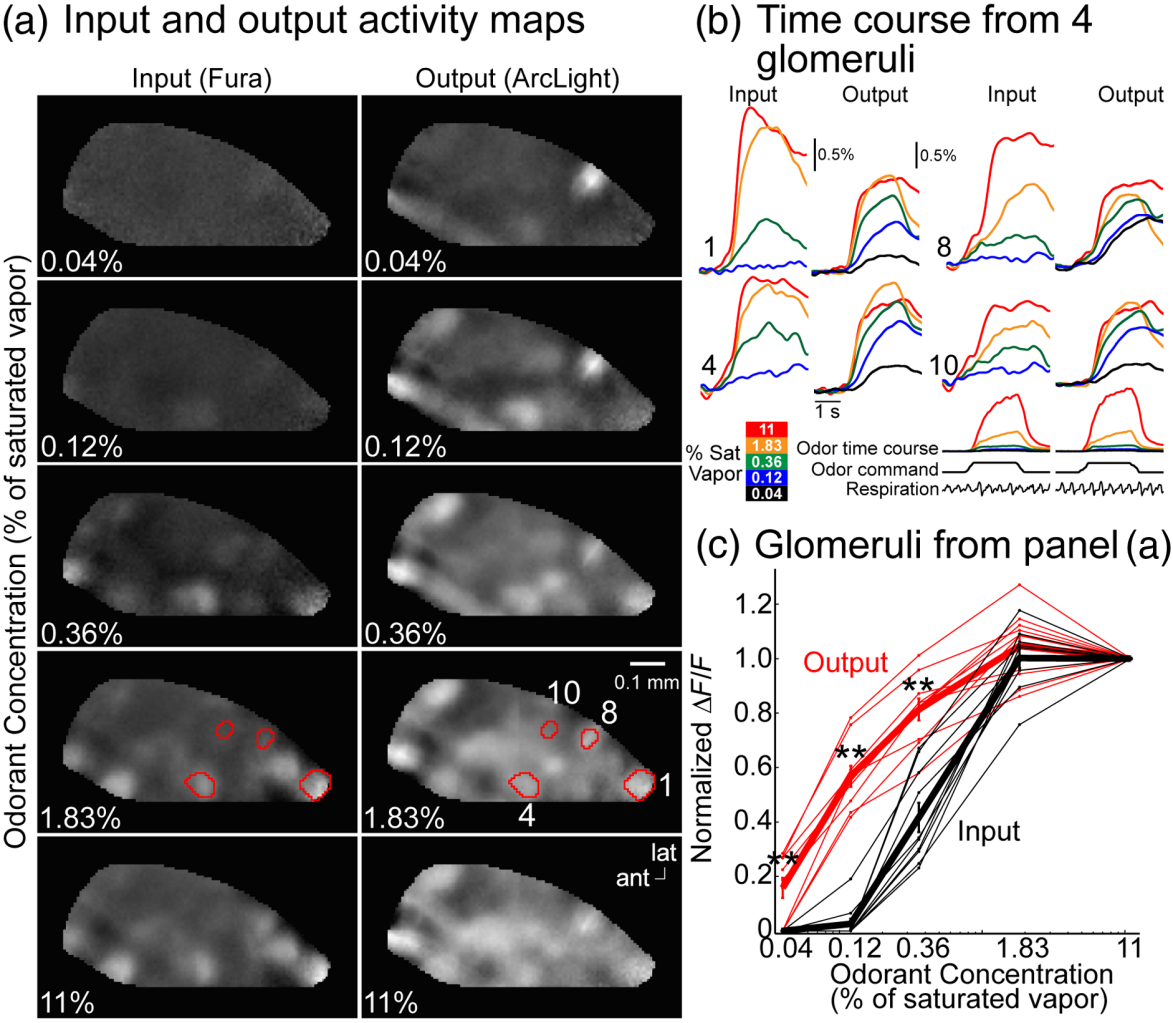
(a) Frame subtraction analysis from ORN (left) and MTC (right) glomeruli at five concentrations of the odor methyl valerate. (b) Fluorescence traces from the regions of interest illustrated in panel (a). (c) Normalized amplitude versus concentration for all the identified glomeruli from the preparation in panel (a).

The ORN and MTC glomerular signals were imaged from anesthetized mice using widefield fluorescence microscopy in response to odors presented across ∼2 log units of concentration. Increasing the concentration of the same odor increased the number of activated ORN glomeruli and altered the combinatorial pattern [Fig. 5(a), input]. In comparison, the spatial pattern of activation measured from the MTC glomeruli changed less across the same concentration changes [Fig. 5(a), output]. The change in concentration dependence can be visualized by comparing the time versus fluorescence traces for ORN and MTC signals innervating different glomeruli [Fig. 5(b)]. The peak ORN and MTC responses were quantified across different concentrations to illustrate the transformation from a highly concentration-dependent ORN input to a MTC response with a less steep concentration-response relationship [Fig. 5(c)]. The different effects of odor concentration on ORN and MTC glomerular maps are qualitatively similar to previous glomerular ORN measurements and single-cell MTC measurements.^21^^,^^148^^,^^167^^,^^179^^,^^203^

The mechanism(s) underlying this transformation remain unclear. Presynaptic inhibition onto the ORNs could dynamically compress the ORN concentration dependence into a narrower range.^134^ However, presynaptic inhibition seems unlikely to be the dominant mechanism since the ORN maps were still concentration-dependent and comparisons of Hill coefficients measured from ORN somata and ORN glomeruli were not significantly different.^118^ A second proposed mechanism involves a global normalization across the glomerular population, a process that can rescale the overall output while maintaining activity across glomeruli at relatively similar levels.^117^^,^^201^ There exist multiple mechanisms that could support a normalization process within the OB. One candidate is a population of interneurons that have long-range lateral connections within the OB and are important in generating stable concentration response relationships in individual mitral cells.^117^^,^^135^^,^^170^^,^^202^

## 9 ArcLight Measurements From TH-Expressing Glomeruli

**Fig. 6:**
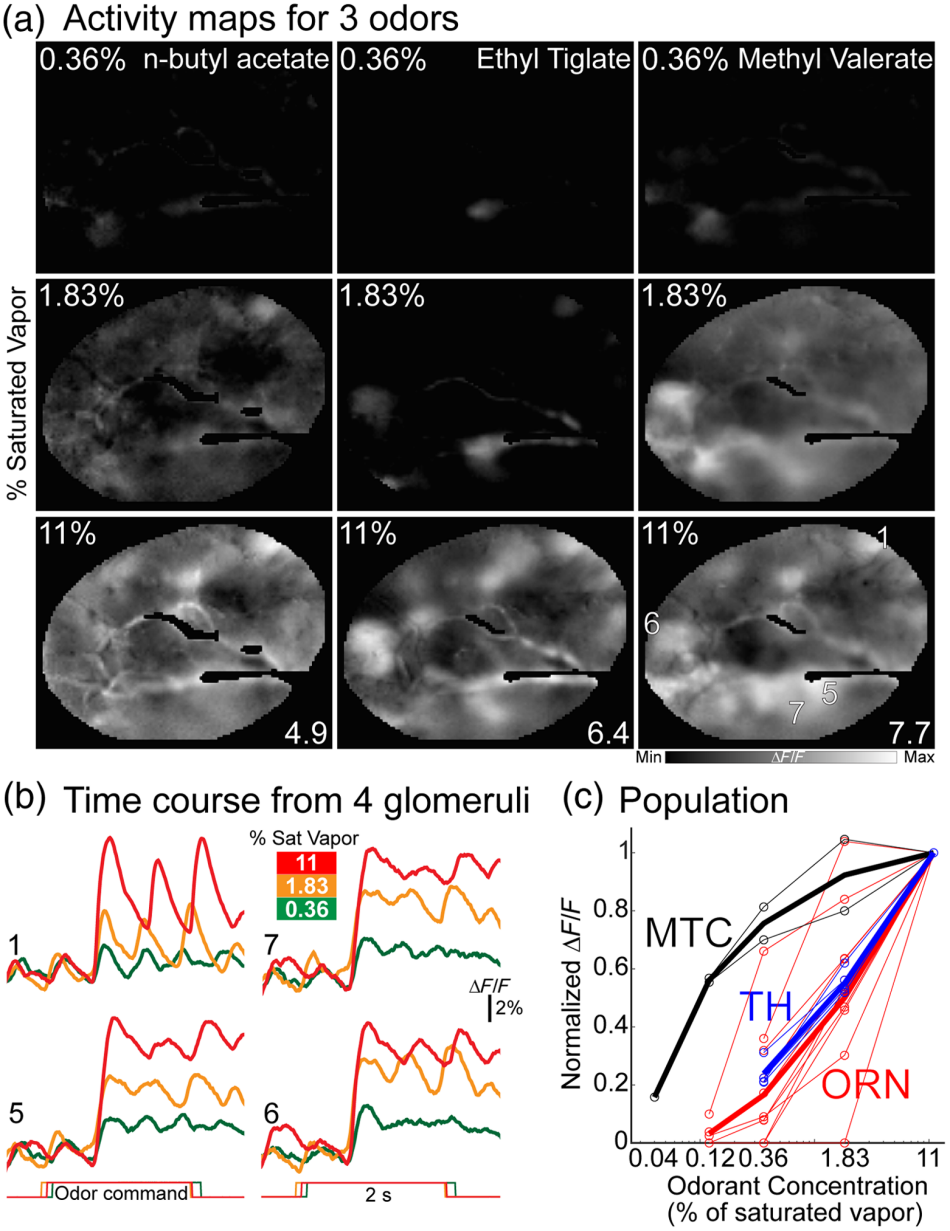
(a) Frame subtraction analysis of different odor-concentration pairings. The odor concentration and maximum ΔF/F for each panel is listed in the top left and bottom right of each panel, respectively. (b) Responses from four glomeruli in response to methyl valerate from panel (a). (c) Normalized response amplitude versus odor concentration for three TH-ArcLight preparations (blue) overlaid with concentration response data from ORN (red) and MTC glomeruli (black).

Measurements from dopaminergic interneuron glomeruli support their role in the broad transmission of concentration-dependent ORN signals.^170^ We performed voltage imaging from the TH interneuron glomeruli to test this model of concentration sensitivity. A Cre-dependent ArcLight-expressing AAV was injected into the OB of TH-Cre transgenic mice [Fig. 3(c)]. Imaging was performed in anesthetized mice in response to different odor-concentration pairings using a widefield fluorescence microscope. A frame subtraction analysis demonstrated that different odors evoked distinct glomerular peaks of activity across the dorsal bulb [Fig. 6(a), columns]. Increasing the concentration of the same odors strongly influenced the amplitude and pattern of the glomerular activity maps [Fig. 6(a), rows]. The impact of concentration on individual glomeruli is illustrated by comparing the fluorescence time course measurements of four TH glomeruli at different concentrations [Fig. 6(b)]. The effect of concentration on TH expressing glomeruli was quantified by measuring their peak response at different concentrations. The TH-ArcLight concentration response relationships were overlaid with the measurements from ORNs and MTCs also carried out using ArcLight [Fig. 6(c), red, blue, and black].^148^^,^^150^^,^^179^ The signals from ORNs and TH glomeruli were similarly concentration-dependent, and the MTC glomeruli changed much less across the same concentration range. These results indicate that the concentration-dependence of TH glomeruli are more similar to ORNs, a result consistent with a previous calcium imaging study from dopamine-expressing glomeruli.^170^

## 10 Two-Photon Imaging with ArcLight in the OB

Brain tissue is highly scattering that poses interpretive challenges for experiments using GEVIs with widefield fluorescence imaging. The degree of scattering is influenced by multiple factors that include the optical properties of the tissue, the imaging optics, and wavelength. In experiments carried out using a photodiode array, light focused on a slice of brain tissue resulted in photons striking multiple detectors separated up to 100  μm.^204^ This results in blurring of signals that will reflect an average of the signals emitted from all the labeled processes.^30^^,^^204^^,^^205^ Therefore, scattering imposes a fundamental limitation on the spatial resolution that can be measured in densely labeled tissue. These issues are exacerbated when imaging from voltage sensors because the signals originate from all parts of a cell. Sparser labeling can facilitate the interpretation of widefield fluorescence measurements by trading off the ability to record from many cells. The value of sparse labeling in voltage imaging is highlighted by experiments in which a voltage sensitive dye is restricted to a single cell, allowing high spatial and temporal resolution measurements from different processes within the same neuron.^30^^,^^206^^–^^208^ Sparser expression has been achieved with GEVIs using low titer AAV transduction and incorporating targeting sequences to restrict expression to parts of a cell.^70^^,^^81^^,^^98^^,^^110^^,^^209^^,^^210^ Another strategy has been to use two-photon microscopy, a laser scanning technique that reduces light scattering by localizing the fluorescence emission to a small spatial volume, reducing the fluorescence that originates from out of focus areas, which substantially improves the lateral and axial resolution that can be achieved in scattering tissue.^211^^,^^212^ Early efforts illustrated the feasibility of incorporating GEVIs with two-photon imaging, although the measurements were limited by signal-to-noise considerations.^213^^,^^214^ Because not all GEVIs work similarly well under widefield fluorescence and two-photon microscopy, it is important to validate each GEVI using both imaging techniques.^96^^,^^215^

**Fig. 7:**
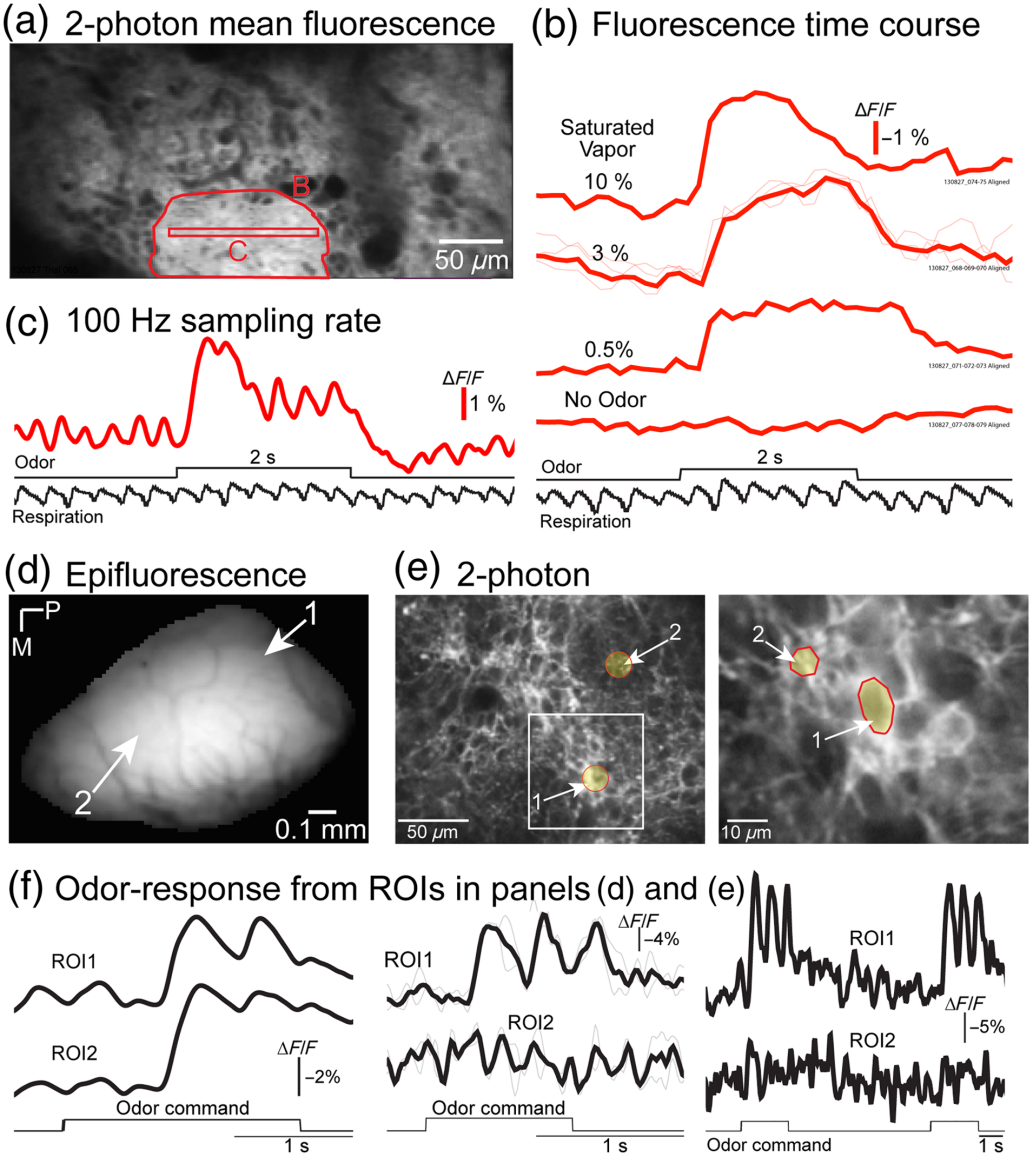
(a) *In vivo* two-photon mean fluorescence following constitutive AAV1 transduction of ArcLight. Odor responses from the regions of interest in panel (a) measured at (b) ∼3  Hz and (c) 100 Hz. Baseline fluorescence from the EMX-ArcLight transgenic mouse measured with (d) widefield fluorescence and (e) two-photon microscopy. The right panel was imaged at a higher magnification zoom from the box in the middle panel. (f) Odor evoked signals from the regions of interest in panels (d) and (e).

We tested the ability of ArcLight in reporting odor-evoked activity in the *in vivo* mouse OB using two-photon microscopy. Imaging was carried out following an injection of a constitutive AAV1 that expressed ArcLight in the OB. In comparison with widefield imaging experiments that used the same AAV1, individual glomeruli and neighboring processes could be clearly resolved in the mean fluorescence using two-photon imaging [Fig. 7(a), red polygon]. In experiments using slow frame rates (∼3  Hz), odor responses could be detected across a range of concentrations, although no respiratory coupling was observed [Fig. 7(b)]. Similar measurements done at a higher sampling rate (100 Hz) from a smaller imaging area [Fig. 7(a), red rectangle] were able to capture respiratory coupled oscillations [Fig. 7(c), taken from red rectangle in panel (a)].

In EMX-ArcLight transgenic mice, widefield fluorescence signals were diffuse, consistent with the presence of ArcLight in deeper layers that contribute out of focus fluorescence [Fig. 7(d), histology in Fig. 3(d)]. In comparison, individual glomeruli and neighboring processes could be clearly visualized using two-photon microscopy [Fig. 7(d) versus Fig. 7(e)]. Odor responses were detected using widefield fluorescence and two-photon imaging [Fig. 7(f), left versus middle panel]. Respiratory coupled oscillations were detected in the widefield fluorescence data that were sampled at 125 Hz and in the two-photon imaging data that were performed at ∼31  Hz [Fig. 7(f), compare left and middle panels]. Using two-photon imaging, odor responses could be detected in small regions of interest that were consistent with the morphology of individual neurons [Fig. 7(e)]. The odor responses were not present everywhere in the imaging field of view, highlighting the higher spatial resolution afforded by the use of two-photon microscopy [Fig. 7(f), compare ROI1 and ROI2 in the middle and right panels]. Therefore, ArcLight reports odor-evoked activity in the mouse OB using both widefield fluorescence and two-photon imaging. These results also highlight that slower two-photon imaging scanning speeds can limit the ability to capture fast events [compare Fig. 7(b) to Figs. 7(c) and 7(f)]. Resonant-based scanners have increased the full frame imaging speed over earlier systems, although additional increases in speed come at a cost of reduced spatial resolution and signal-to-noise since faster scanning shortens the laser dwell time, which shortens the time during which emitted photons can be measured. Recent advances in the use of acousto-optic modulators that allow for arbitrary imaging patterns at high temporal resolution offer a promising alternative to current scanners to capture higher frequency neuronal signaling.^98^^,^^110^^,^^216^

## 11 Interpretation of the Population Measurements

The interpretation of the optical signals measured using ArcLight is complicated and depends on the targeting strategy and imaging modality. Although ArcLight was targeted to specific OB cell populations in these experiments, the expression was present in many neurons. Consider OMP-ArcLight, which is less impacted by the complications of light scattering because the fluorescence was restricted to ORN glomeruli on the dorsal surface [Fig. 3(a)]. Ignoring the impact of scattered fluorescence from lateral glomeruli, the signal from each glomerulus reflects the input from many individual ORNs and therefore the measurement from a glomerulus is an average of all the active and inactive labeled neurons. If 1000 asynchronously active neurons in a volume of tissue express a GEVI, the ΔF from any single-active neuron will be overshadowed by the fluorescence of the inactive cells and reduced proportionally by the number of inactive neurons. Moreover, any hyperpolarized neurons would fluoresce in the opposite direction of the depolarized neurons, which would further diminish the averaged fluorescence change. Therefore, the signal from a single-action potential would depend on the number of synchronously active and inactive cells, and therefore measurements of action potential activity in population measurements likely requires precise temporal synchronization of spiking activity across many of the labeled neurons. Indeed, OB spiking activity is highly coupled to respiration in anesthetized mice, which provides a mechanism to tightly synchronize action potentials.^154^^,^^183^ However, the maximum ΔF/F response of ArcLight is only measured with a constant depolarization of 10 ms or longer.^63^^,^^91^ Since the voltage-dependence range of ArcLight includes subthreshold potentials, the population signals measured from ArcLight likely reflect some complex mixture of subthreshold and action potential activity. The use of GEVIs tuned to different voltage-ranges would allow for a clearer interpretation as to whether a population signal primarily reflects hyperpolarizing, subthreshold, or voltage changes.

Recent efforts to shape the voltage-response relationships of some GEVIs illustrate that this property can be modified with appropriate mutations.^74^^,^^82^^,^^92^ Ultimately there may not be a perfect GEVI for all applications, but a series of differently tuned GEVIs optimized to measure different kinds of voltage signals (e.g., subthreshold versus action potential events), analogous to the current strategies used for GECI development.^65^^,^^66^^,^^69^^,^^82^^,^^217^ For example, a GEVI with high sensitivity and instantaneous onset kinetics but with a voltage-dependence tuned to action potentials and a slow decay rate would be ideal for two-photon imaging on commonly available systems. A slower decay rate would extend the integration time to measure an action potential at a cost of losing the ability to resolve individual action potentials from exceptionally fast spiking neurons. The parallel development of sensitive GEVIs available in a range of fluorophores will facilitate the ability to perform simultaneous imaging from multiple sensors in a single imaging plane allowing for measurements of subthreshold and action potential activity, from different cell types or different neuronal signaling molecules.^82^^,^^107^

## 12 Conclusions

Here we reviewed recent work demonstrating that the GEVI ArcLight can be used to measure glomerular signals from odor-evoked activity in the mouse OB from different cell types using widefield fluorescence and two-photon imaging. The faster temporal dynamics of ArcLight more precisely captured the respiratory coupled dynamics of glomerular activity when compared to GCaMP3 and GCaMP6f (Fig. 2). The ArcLight measurements illustrated that concentration coding is transformed across the OB circuit. These voltage imaging experiments validate work using GECIs demonstrating that a highly concentration-dependent ORN input signal is transformed into a more concentration invariant output response.^148^ Moreover, the glomerular output still maintained some sensitivity to concentration changes, indicating that concentration differences are still represented. In principle, such a coding scheme would allow downstream targets to recognize odors in a concentration invariant manner while still maintaining the ability to distinguish between concentration differences based on the output pattern of the bulb.

The growth of the number of laboratories developing and improving GEVIs over the last decade suggests an optimistic trajectory for making GEVIs easier to use *in vivo*, although the increasing number of those that are available complicates the choice of any particular one without clear comparisons of how they work in the *in vivo* brain. Moreover, the optimal GEVI choice likely depends on region-specific physiological parameters (e.g., varying spike widths and the overall balance of depolarization and hyperpolarization). Given that caveat, we propose that one potentially useful future goal is to have a model testing system for *in vivo* measurements by which all GEVIs could be screened for their ability to report population and single-cell activity using different microscopy techniques. We propose that the OB may be such a useful model brain area due to it containing dense neuropil within glomeruli as well as single cells that surround glomeruli that are easily accessible on the dorsal surface. Together, such developments and improvements will facilitate the uptake of GEVIs by the broader community to advance our understanding of the brain.

## Data Availability

All data described in this manuscript are available from the authors upon request.
